# Supplementary material for: A new class of antibodies that overcomes a steric barrier to cross-group neutralization of influenza viruses
Source: PLoS Biol. 2023 Dec 21;21(12):e3002415. doi: 10.1371/journal.pbio.3002415 (PMC10734940; doi:10.1371/journal.pbio.3002415)
Supplement: S1 Table — (PDF) [file pbio.3002415.s001.pdf]

Table S1 Data collection and refinement statistics

	K03.28-A/California/7/2009 (H1N1) (NYMC-X181) (H1-X181) Fab-HA head complex	S8V1-172-A/Sydney/05/1997(H3N2) Fab-HA head complex
**PDB ID**	7TRH	7TRI
**Data Collection**	APS 24-ID-C	APS 24-ID-C
Number of datasets	1	1
Resolution, Å	46.80-3.00 (3.11-3.0)	47.35-3.60 (3.73-3.60)
Wavelength (Å)	0.9791	0.9791
Space Group	P 1 21 1	P 32 2 1
Unit cell dimensions (a, b, c), Å	75.83 43.65 96.91	94.7 94.7 248.84
Unit cell angles (α, β, γ) °	90.00 105.03 90.00	90.00 90.00 120.00
I/σ	14.93 (2.10)	12.72 (3.04)
Rmeas	0.06 (0.54)	0.06 (0.46)
Rpim	0.04 (0.38)	0.046 (0.32)
Rmerge, %	0.04 (0.38)	0.045 (0.32)
CC*	1.00 (0.93)	1.00 (0.91)
CC½	1.00 (0.77)	1.00 (0.71)
Completeness, %	98.33 (97.87)	99.46 (99.87)
Number of reflections	24401 (2401)	30957 (3053)
Redundancy	2.0 (1.9)	2.0 (2.0)
**Refinement**		
Number of reflections:		
Working	12440 (1241)	15605 (1543)
Free	623 (62)	789 (86)
Rwork, %	20.20 (31.45)	23.73 (31.01)
Rfree, %	24.32 (35.44)	26.66 (34.98)
Ramachandran plot, % (favored, disallowed)	93.42 (0.16)	93.42 (0.14)
Rmsd bond lengths, Å	0.003	0.003
Rmsd bond angles, °	0.64	0.61
Average B-factor	78.52	119.17

Rmerge, Σh,k,lΣi|Ii(hkl) – <I(hkl)>|/Σh,k,lΣiIi(hkl), where I is an intensity that is observed i times; I/σ, signal-to-noise ratio (average observed intensity divided by average standard deviation of the observed intensity); Rwork, Σh,k,l||Fobs| – |Fcalc||/Σh,k,l|Fobs|, where h, k, l covers the "working set" of observed structure factor amplitude (Fobs) reflections used in refinement (total reflections minus the test set) and Fcalc is the calculated structure factor amplitude; Rfree, calculated as for Rwork but on 5% of data excluded prior to refinement. Values in parentheses refer to highest-resolution shell. Related to Experimental Procedures.
