# Supplementary material for: A new class of antibodies that overcomes a steric barrier to cross-group neutralization of influenza viruses
Source: PLoS Biol. 2023 Dec 21;21(12):e3002415. doi: 10.1371/journal.pbio.3002415 (PMC10734940; doi:10.1371/journal.pbio.3002415)
Supplement: S1 Fig — (A) Microneutralization titers for antibody K03.28, S8V1-172, S5V2-107, and S8V2-124. All values are in μg/ml. (B) We note there are 8 amino acid differences between the HA of A/Moscow/10/1999(H3N2) (GenBank DQ487341) used in binding assays (Fig 3) and the A/Moscow/10/1999(H3N2)(X-137) reassortant virus used in microneutralization assays (GenBank CY121381). Their positions are shown in red sticks (145K/N, 158E/K,159N/Y, 190D/V, 194V/L 196V/T, 226I/V, and 246N/K) in the structure of S8V1-172 complexed with the A/Sydney/05/1997(H3N2) HA head domain. K03.28 modeled in through superposition upon their respective HA head domains (not shown). Manual inspection suggests that a subset of mutations found in A/Moscow/10/1999(H3N2) would disrupt HA-Fab contacts at positions 145 and 159 and introduce an unfavorable contact at position 190. Figure data are in S1 Data. (PDF) [file pbio.3002415.s002.pdf]

A

Strain Name	K03.28	S8V1-172	S5V2-107	S8V2-124
A/USSR/90/1977 X-67 (H1N1)	25	>50	>50	0.781
A/Chile/01/1983 (H1N1)	>50			
A/Beijing/262/1995 (H1N1)	>50			
A/Solomon Islands/03/2006 (H1N1)	0.195			
A/California/04/2009 (H1N1)		>50	>50	>50
A/California/07/2009 NYMC X-181 (H1N1)		0.138	<0.049	<0.049
A/Michigan/45/2015 (H1N1)		>50	25	1.563
A/Aichi/02/1968 (H3N2)	>50			
A/Victoria/03/1975 X-47 (H3N2)	>50			
A/Philippines/2/1982 (H3N2)	>50	>50	>50	50
A/Shanghai/11/1987 X-99a (H3N2)	12.5			
A/Johannesburg/33/1994 X-123a (H3N2)	<0.049	0.195	<0.049	<0.049
A/Moscow/10/1999 X-137 (H3N2)	0.069	<0.049	<0.049	<0.049
A/Wisconsin/67/2005 (H3N2)	>50			
A/Kansas/14/2017 NYMC X-327 (H3N2)		>50	>50	>50

B

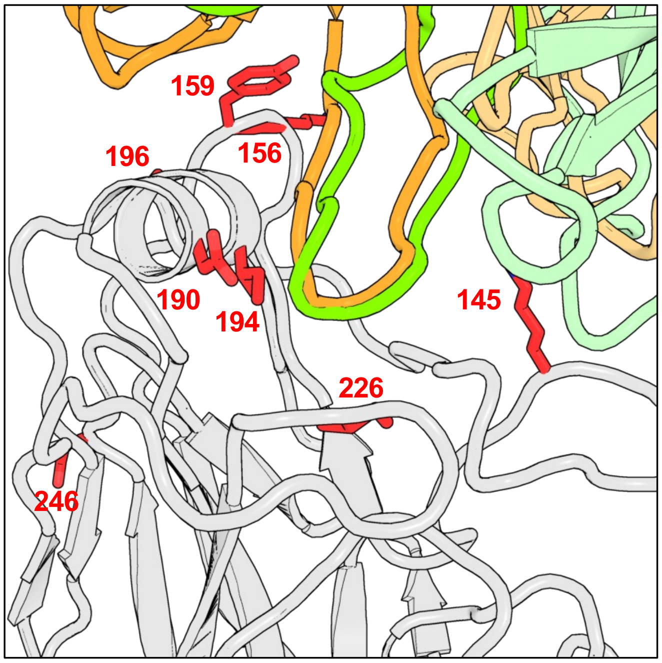

S1. Microneutralization data and differences between A/Moscow/10/1999(H3N2) isolates. A. Microneutralization titers for antibody K03.28, S8V1-172, S5V2-107 and S8V2-124. All values are in µg/ml. **B.** We note there are eight amino acid differences between the HA of A/Moscow/10/1999(H3N2) (GenBank DQ487341) used in binding assays (Figure 3) and the A/Moscow/10/1999(H3N2)(X-137) reassortant virus used in microneutralization assays (GenBank CY121381). Their positions are shown in red sticks (145K/N, 158E/K,159N/Y, 190D/V, 194V/L 196V/T, 226I/V, and 246N/K) in the structure of S8V1-172 complexed with the A/Sydney/05/1997(H3N2) HA head domain. K03.28 modeled in through superposition upon their respective HA head domains (not shown). Manual inspection suggests that a subset of mutations found in A/Moscow/10/1999(H3N2) would disrupt HA-Fab contacts at positions 145 and 159 and introduce an unfavorable contact at position 190.
