# Supplementary material for: A new class of antibodies that overcomes a steric barrier to cross-group neutralization of influenza viruses
Source: PLoS Biol. 2023 Dec 21;21(12):e3002415. doi: 10.1371/journal.pbio.3002415 (PMC10734940; doi:10.1371/journal.pbio.3002415)
Supplement: S2 Fig — A view of antibody contacts (sticks) with the RBS oriented identically to Fig 2. Antibodies and HA heads are colored the same as Fig 2 (H1 head in cyan, H3 gray). The E-G-W motifs of K03.28 (green) and S8V1-172 (orange) are compared to an A-G-W in present in antibody C05 [11] (magenta; PDB 4FP8). (PDF) [file pbio.3002415.s003.pdf]

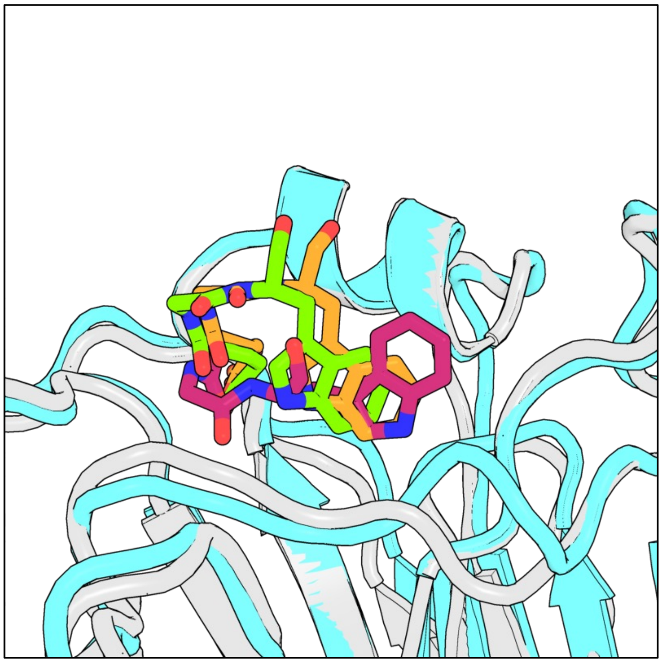

Figure S2. Antibodies K03.28 and S8V1-172 contact the receptor binding site differently from C05. A view of antibody contacts (sticks) with the RBS oriented identically to Figure 2. Antibodies and HA heads are colored the same as Figure 2 (H1 head in cyan, H3 gray). The E-G-W motifs of K03.28 (green) and S8V1-172 (orange) are compared to an A-G-W in present in antibody C05[11] (magenta; PDB 4FP8).
