# Supplementary material for: A new class of antibodies that overcomes a steric barrier to cross-group neutralization of influenza viruses
Source: PLoS Biol. 2023 Dec 21;21(12):e3002415. doi: 10.1371/journal.pbio.3002415 (PMC10734940; doi:10.1371/journal.pbio.3002415)
Supplement: S3 Fig — Gene utilization and CDR3 sequences of the 10 antibodies with an E-G-W motif (A) or those with similar patterns of HA reactivity (B). Amino acids in common with the E-G-W motif are bolded. Sequence logos below each panel were produced from an initial alignment of all antibody HCDR3s that was then subdivided into 2 alignments, 1 specific for each panel, without realignment. This was done to allow for comparisons between panels A and B. Gaps in the sequence logo are sites of length variation. Sequence logos were produced with WebLogo [46]. Figure data are in S1 Data. (PDF) [file pbio.3002415.s004.pdf]

A

Donor	Antibody	IGHV	IGHD	IGHJ	HCDR3 Sequence	HCDR3 length	IgLV	IGLJ	LCDR3	LCDR3 Length
KEL03	K03.28	IGHV3-48	IGHD3-10	IGHJ6	CARLLDSIVWG**EGW**YYGMDVW	19	IGLV3-21	IGLJ2 or IGLJ3	CQVWDTSSGPPHVIF	13
S5	S5V2-42	IGHV3-15	IGHD2-15	IGHJ6	CTTENVVVG**EGW**YYGMDVW	17	IGLV2-14	IGLJ2 or IGLJ3	CSSYGSITTLDVVF	12
S5	S5V2-79	IGHV3-15	IGHD2-15	IGHJ6	CTTENVVVG**EGW**FNGMDVW	17	IGLV2-14	IGLJ2 or IGLJ3	CSSYTNTNTLDVVF	12
S8	S8V1-137	IGHV4-59	IGHD3-3	IGHJ5 or IGHJ6	CARHDVVFG**EGW**YYGLDIW	17	IGKV3-11	IGKJ4	CQQDSNWLTF	8
S8	S8V1-144	IGHV4-59	IGHD2-2	IGHJ6	CARDDIVVG**EGW**YNGMDVW	17	IGKV3-11	IGKJ4	CQHGSTWPTF	8
S8	S8V1-172	IGHV4-59	IGHD2-2	IGHJ6	CARDDIVIG**EGW**YHGMDLW	17	IGKV3-11	IGKJ4	CQHRSTWVTF	8
S8	S8V2-40	IGHV4-61	IGHD3-10	IGHJ3 or IGHJ6	CARLSMVKG**EGW**FDGLDVW	17	IGKV3-20	IGKJ4	CQHYDGSLTF	8
S8	S8V2-67	IGHV4-59	IGHD2-2	IGHJ6	CARDDIVIG**EGW**YHGMDLW	17	IGKV3-11	IGKJ4	CQHRSTWVTF	8
S8	S8V2-112	IGHV4-59	IGHD3-10	IGHJ6	CARVSMVQG**EGW**YYGMDVW	17	N.R.	N.R.	N.R.	N.R.
S8	S8V2-113	IGHV4-59	IGHD3-10	IGHJ6	CARVSMVLG**EGW**FHGMDVW	17	IGLV3-1	IGLJ3	CQAWDSNTVVF	9
S8	S8V2-117	IGHV4-59 or IGHV4-61	IGHD3-10	IGHJ6	CARISMVLG**EGW**YHGMDVW	17	N.R.	N.R.	N.R.	N.R.

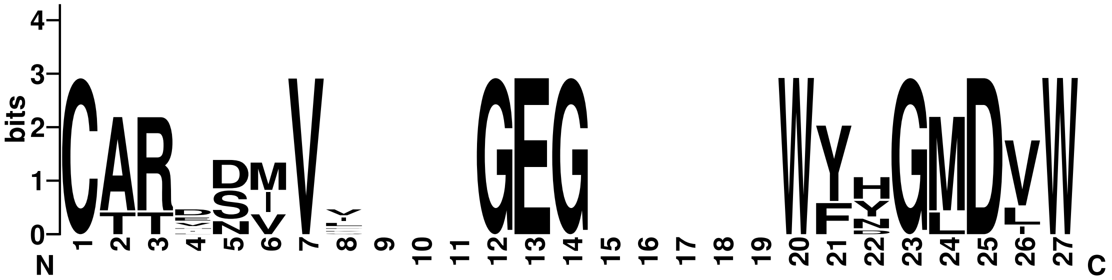

B

Donor	Antibody	IGHV	IGHD	IGHJ	HCDR3 Sequence	HCDR3 length	IgLV	IGLJ	LCDR3	LCDR3 Length
S5	S5V2-28	IGHV3-15	IGHD2-15	IGHJ3 or IGHJ6	CATENVVVG**E**H**W**YRGFDVW	17	IGLV2-14	IGLJ2 or IGLJ3	CSSYTNTNTLDVVF	12
S5	S5V2-34	IGHV3-15	IGHD2-15	IGHJ6	CTTENVVEG**E**H**W**YHGLDVW	17	IGLV2-14	IGLJ2 or IGLJ3	CSSYAXTNTLXVVF	12
S5	S5V2-35	IGHV3-15	IGHD2-15	IGHJ6	CTTENVVVG**E**S**W**YYGLDVW	17	IGLV2-14	IGLJ2 or IGLJ3	CSSYTSISTLDVVF	12
S5	S5V2-44	IGHV3-15	IGHD2-15	IGHJ6	CTTENVVVG**E**H**W**YHGLDVW	17	IGLV2-14	IGLJ2 or IGLJ3	CSSYTNMNTLDVVF	12
S5	S5V2-46	IGHV3-15	IGHD2-15	IGHJ6	CCTENVVVG**E**N**W**YYGLDVW	17	IGLV2-14	IGLJ2 or IGLJ3	CCSYSSIITLDVVF	12
S5	S5V2-51	IGHV3-15	IGHD2-15	IGHJ6	CTTENVVVG**E**N**W**YHGLDVW	17	IGLV2-14	IGLJ2 or IGLJ3	CSSYTNMNTLDVVF	12
S5	S5V2-67	IGHV3-48	IGHD4-17	IGHJ6	CARFDEDA**EG**YYGMDVW	15	IGKV1-5	IGKJ1	CQQYNAYWTF	8
S5	S5V2-73	IGHV4-61	IGHD5-18	IGHJ6	CARDMLHFDRYSYGYSPDYYYYGMDVW	25	IGLV2-14	IGLJ2 or IGLJ3	CSSYTNINTLDVVF	12
S5	S5V2-74	IGHV3-15	IGHD2-15	IGHJ6	CTTEDVVVG**E**N**W**YNGMDVW	17	IGLV2-14	IGLJ2 or IGLJ3	CSSYTSSSTLDVVF	12
S5	S5V2-77	IGHV3-30	IGHD2-21	IGHJ4	CAKAGNAGWECGSDCYEGDYW	19	IGLV2-14	IGLJ1	CSSHASSSPYVF	10
S5	S5V2-81	IGHV3-48	IGHD4-17	IGHJ6	CARFDEDA**EG**YYGMDVW	15	IGKV1-5	IGKJ1	CQQYNFYWTF	8
S5	S5V2-82	IGHV3-15	IGHD2-15	IGHJ5 or IGHJ6	CITENVVVG**E**H**W**YNGFDVW	17	IGLV2-14	IGLJ2 or IGLJ3	CSAYTTTNTLDVVF	12
S5	S5V2-86	IGHV3-15	IGHD2-15	IGHJ6	CTTENVVVG**E**H**W**YHGMDVW	17	IGLV2-14	IGLJ2 or IGLJ3	CSSYTNINTLDVVF	12
S5	S5V2-98	IGHV3-15	IGHD2-15	IGHJ6	CTTENVVVG**E**H**W**FYGLDIW	17	IGLV2-14	IGLJ2 or IGLJ3	CSSYTTSGTLDVVF	12
S5	S5V2-99	IGHV3-30	IGHD3-16	IGHJ6	CARADEDV**EG**YYGMDVW	15	IGKV1-5	IGKJ2	CQQYNNYPHTF	9
S5	S5V2-100	IGHV3-15	IGHD2-15	IGHJ6	CTTENVVVG**E**H**W**YHGLDVW	17	IGLV2-14	IGLJ2 or IGLJ3	CSSYTTATTLDVVF	12
S5	S5V2-103	IGHV3-15	IGHD2-15	IGHJ6	CTTENVVVG**E**H**W**YHGLDVW	17	IGLV2-14	IGLJ2 or IGLJ3	CSSYTTATTLDVVF	12
S5	S5V2-107	IGHV3-15	IGHD2-15	IGHJ6	CTTENVVVG**E**H**W**FNGMDVW	17	IGLV2-14	IGLJ2 or IGLJ3	CSSYTNINTLDVIF	12
S5	S5V2-112	IGHV3-48	IGHD5-12	IGHJ6	CATSRPSGYDG**EG**FFYYYYGMDVW	22	IGKV1-9	IGKJ3	CQQLNSPFTF	8
S5	S5V2-113	IGHV3-48	IGHD4-17	IGHJ3 or IGHJ6	CARFDEDA**EG**HYGMDVW	15	IGKV1-5	IGKJ1	CQQYNVYWTF	8
S5	S5V2-116	IGHV3-48	IGHD4-17	IGHJ6	CARFDEDA**EG**YYGMDVW	15	IGKV1-5	IGKJ1	CQQYNAYWTF	8
S8	S8V1-126	IGHV1-69 or IGHV1-69D	IGHD3-10	IGHJ5	CATTHPPRG**EG**VVLGIIEKGFDPW	22	IGKV1-39 or IGKV1D-39	IGLJ2 or IGLJ3	CQQTFGAPYNF	9
S8	S8V1-140	IGHV3-33	IGHD5-12	IGHJ6	CARTDMDV**EG**YYGMDVW	15	IGKV1-5	IGKJ1	CQQYYTYSWTF	9
S8	S8V1-170	IGHV3-33	IGHD5-12	IGHJ6	CARTDMDV**EG**YYGMDVW	15	IGKV1-5	IGKJ1	CQQYYTYSWTF	9
S8	S8V2-14	IGHV1-69 or IGHV1-69D	IGHD3-3	IGHJ5	CARSHPPGG**EG**VVFGILEKGFDPW	22	IGKV1-39 or IGKV1D-39	IGKJ2	CQQTYGSPYTF	9
S8	S8V2-56	IGHV3-13	IGHD3-9	IGHJ6	CAGGDYDI**EG**YYGMDVW	15	IGKV1-5	IGKJ4	CQQYYSYPLTF	9
S8	S8V2-58	IGHV3-23	IGHD6-19	IGHJ4	CAKEDIAVA**G**YYFDYW	14	IGKV3-11	IGKJ2	CQQRSNWPRCSF	10
S8	S8V2-88	IGHV3-33	IGHD2-2	IGHJ6	CARIDMDV**EG**YYAMDVW	15	IGKV1-5	IGKJ1	CQHYDHYSWTF	9
S8	S8V2-124	IGHV3-33	IGHD2-2	IGHJ6	CGRLDMDV**EG**YYGMDVW	15	IGKV1-5	IGKJ1	CQHYDHYSWTF	9

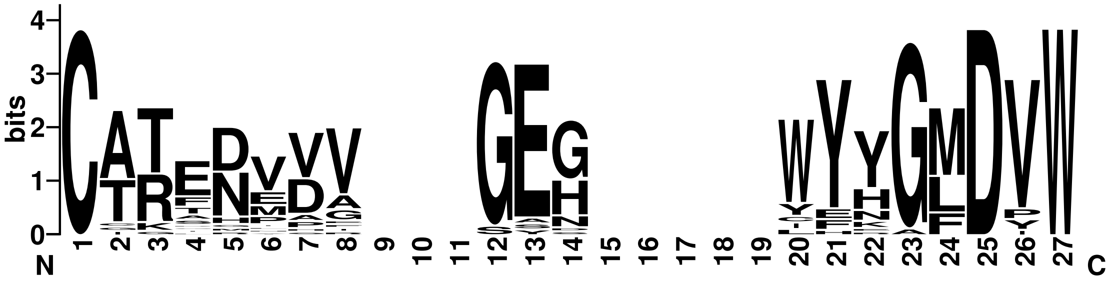

Figure S3. Antibody V(D)J usage and CDR3 sequences. Gene utilization and CDR3 sequences of the 10 antibodies with a E-G-W motif **(A)** or those with similar patterns of HA reactivity **(B)**. Amino acids in common with the E-G-W motif are bolded. Sequence logos below each panel were produced from an initial alignment of all antibody HCDR3 that was then subdivided into two alignments, one specific for each panel, without realignment. This was done to allow for comparisons between panel A and B. Gaps in the sequence logo are sites of length variation. Sequence logos were produced with WebLogo[46].
