# Supplementary material for: A new class of antibodies that overcomes a steric barrier to cross-group neutralization of influenza viruses
Source: PLoS Biol. 2023 Dec 21;21(12):e3002415. doi: 10.1371/journal.pbio.3002415 (PMC10734940; doi:10.1371/journal.pbio.3002415)
Supplement: S5 Fig — The broadly binding, head interface directed S5V2-29 [22] was used as a positive control and an influenza B specific, RBS directed antibody, CR8033 [25], as a negative control for influenza A isolates. Data points represent the average of 3 technical replicates. The standard error of the mean is shown for each point. KDs were calculated from the curves fit to these data points. Figure data are in S2 Data. (PDF) [file pbio.3002415.s006.pdf]

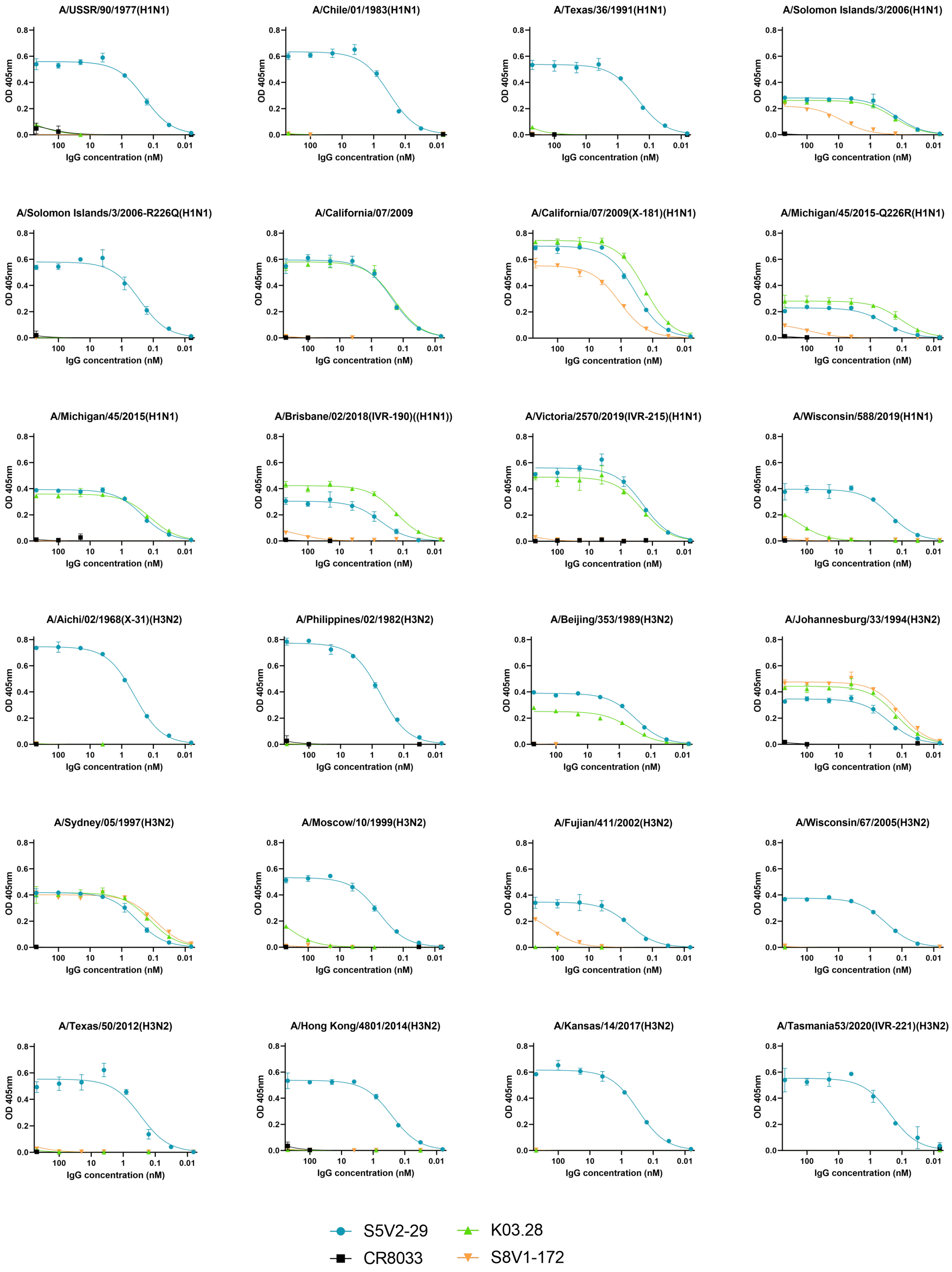

Figure S5. ELISA titrations of antibodies on HA coated plates. The broadly binding, head interface directed S5V2-29[22] was used as a positive control and an influenza B specific, RBS directed antibody, CR8033[25], as a negative control for influenza A isolates. Data points represent the average of three technical replicates. The standard error of the mean is shown for each point. KDs were calculated from the curves fit to these data points.
