# Supplementary material for: A new class of antibodies that overcomes a steric barrier to cross-group neutralization of influenza viruses
Source: PLoS Biol. 2023 Dec 21;21(12):e3002415. doi: 10.1371/journal.pbio.3002415 (PMC10734940; doi:10.1371/journal.pbio.3002415)
Supplement: S6 Fig — HA R226 but not Q226 mediate contacts with the HCDR3 E-G-W motif. Structures of the A/California/04/2009(H1N1) (Q226) HA head domain (light blue, PDB 3UBE) and S8V1-172 Fab-HA complex are superposed on the HA head of domain of the K03.28- A/California/07/2009(X-181)(H1N1) (R226) complex (cyan). The HA is not shown for S8V1-172 complex. The HCDR3 E-G-W motif of K03.28 (green) and S8V1-172 (orange) are shown in sticks. HA R226 and Q226 are shown in sticks. A hydrogen bond to the carbonyl of the E is shown in black dashed lines. Panel A is in the same orientation as Fig 1 and Panel B is in the same orientation as Fig 2. (PDF) [file pbio.3002415.s007.pdf]

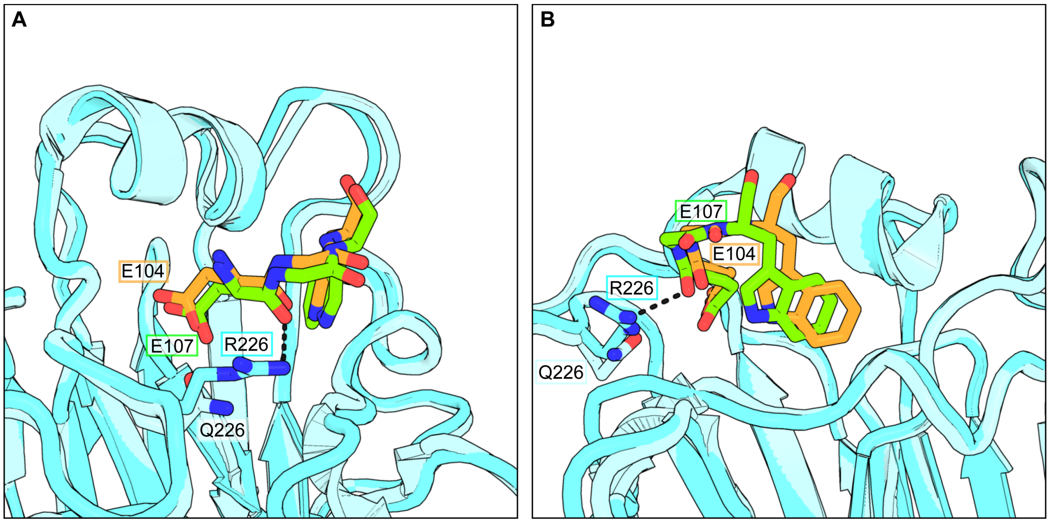

Figure S6. R226 contacts HCDR3. HA R226 but not Q226 mediate contacts with the HCDR3 E-G-W motif. Structures of the A/California/04/2009(H1N1) (Q226) HA head domain (light blue, PDB 3UBE) and S8V1-172 Fab-HA complex are superposed on the HA head of domain of the K03.28- A/California/07/2009(X-181)(H1N1) (R226) complex (cyan). The HA is not shown for S8V1-172 complex. The HCDR3 E-G-W motif of K03.28 (green) and S8V1-172 (orange) are shown in sticks. HA R226 and Q226 are shown in sticks. A hydrogen bond to the carbonyl of the E is shown in black dashed lines. **Panel A** is in the same orientation as Figure 1 and **Panel B** is in the same orientation as Figure 2.
