# Supplementary material for: A new class of antibodies that overcomes a steric barrier to cross-group neutralization of influenza viruses
Source: PLoS Biol. 2023 Dec 21;21(12):e3002415. doi: 10.1371/journal.pbio.3002415 (PMC10734940; doi:10.1371/journal.pbio.3002415)
Supplement: S7 Fig — Clonal antibody lineages were identified in our dataset using Cloanalyst [36]. The unmutated common ancestors were inferred using Cloanalyst [36] and used for subsequent studies. Clonograms for the S8V1-172 and S5V2-107 lineages are shown. Figure data are in S1, S3 and S4 Datas. (PDF) [file pbio.3002415.s008.pdf]

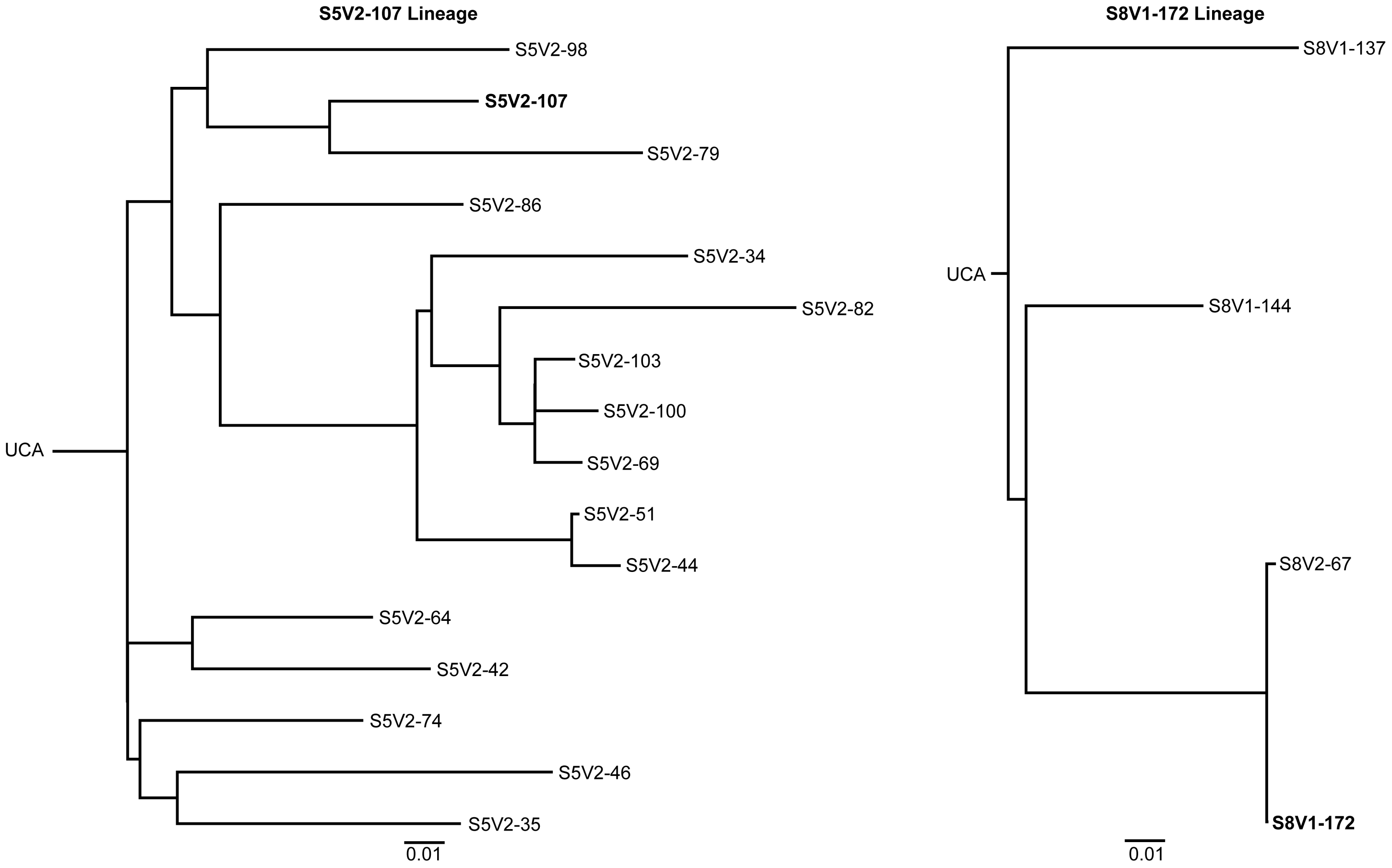

Figure S7. S8V1-172 and S5V2-107 clonal antibody lineages. Clonal antibody lineages were identified in our dataset using Cloanalyst[36]. The unmutated common ancestors were inferred using Cloanalyst[36] and used for subsequent studies. Clonograms for the S8V1-172 and S5V2-107 lineages are shown.
