# Supplementary material for: A new class of antibodies that overcomes a steric barrier to cross-group neutralization of influenza viruses
Source: PLoS Biol. 2023 Dec 21;21(12):e3002415. doi: 10.1371/journal.pbio.3002415 (PMC10734940; doi:10.1371/journal.pbio.3002415)
Supplement: S8 Fig — The broadly binding, head interface directed S5V2-29 [22] was used as a positive control and an influenza B specific, RBS directed antibody, CR8033 [25], as a negative control for influenza A isolates. (A) Data points represent the average of 3 technical replicates. The standard error of the mean is shown for each point. (B) KDs were calculated from the curves fit to these data points and are included below. Figure data are in S1 and S5 Datas. (PDF) [file pbio.3002415.s009.pdf]

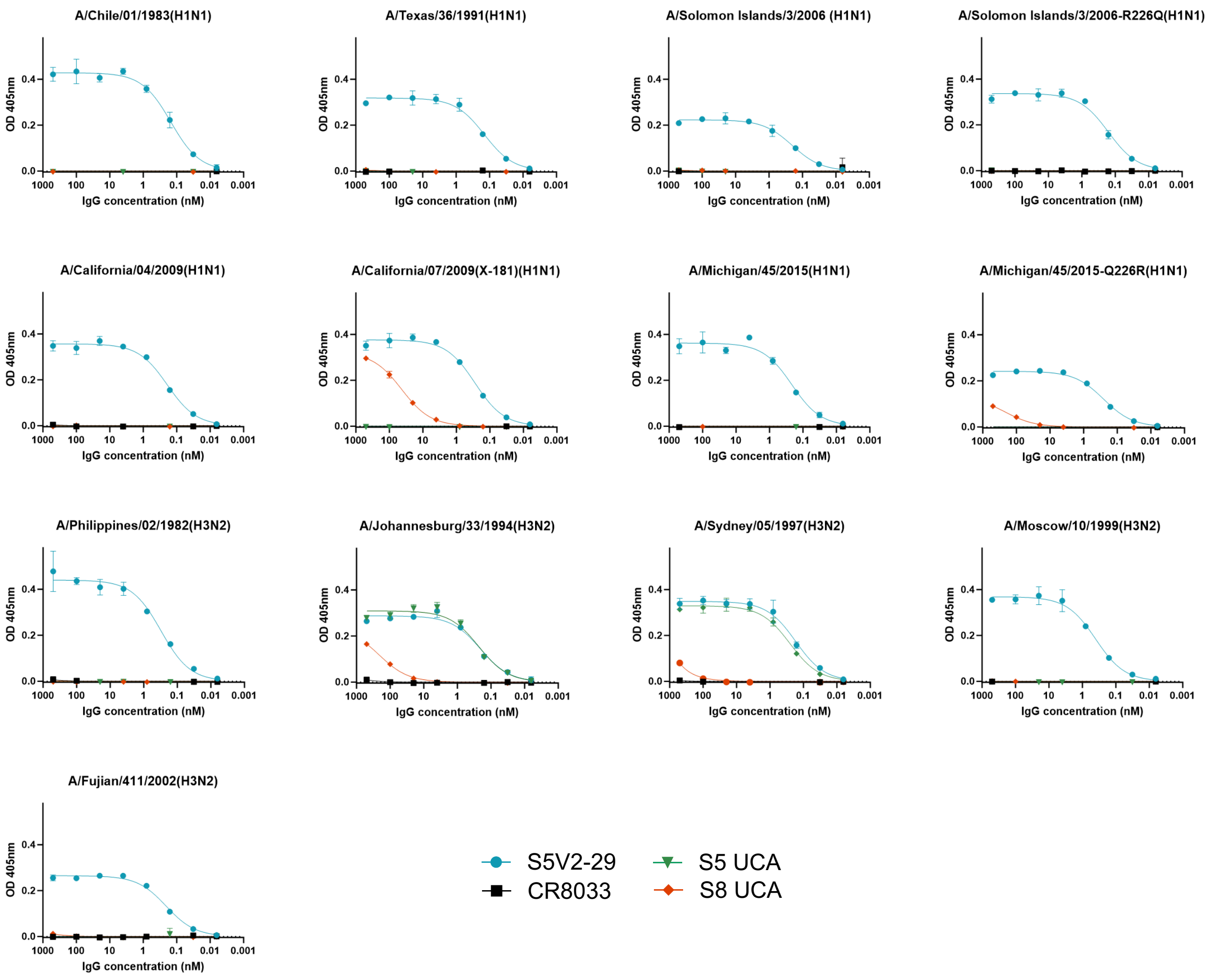

			Antibody			
		Pos. 226	S5 UCA	S8 UCA	CR8033	S5V2-29
H1N1	A/Chile/01/1983	Q	>100	>100	>100	0.15
	A/Texas/36/1991	Q	>100	>100	>100	0.14
	A/Solomon Islands/3/2006	R	>100	>100	>100	0.20
	A/Solomon Islands/3/2006-R226Q	Q	>100	>100	>100	0.20
	A/California/04/2009	Q	>100	>100	>100	0.19
	A/California/07/2009(X-181)	R	>100	43	>100	0.28
	A/Michigan/45/2015	Q	>100	>100	>100	0.21
	A/Michigan/45/2015-Q226R	R	>100	>100	>100	0.25
H3N2	A/Philippines/02/1982	L	>100	>100	>100	0.31
	A/Johannesburg/33/1994	Q	0.23	>100	>100	0.20
	A/Sydney/05/1997	I	0.25	>100	>100	0.17
	A/Moscow/10/1999	V	>100	>100	>100	0.40
	A/Fujian/411/2002	V	>100	>100	>100	0.21

Kd in nM				
<1	1-5	5-25	25-100	>100

Figure S8. ELISA titrations and affinities of antibody UCAs. The broadly binding, head interface directed S5V2-29[22] was used as a positive control and an influenza B specific, RBS directed antibody, CR8033[25], as a negative control for influenza A isolates. **A.** Data points represent the average of three technical replicates. The standard error of the mean is shown for each point. **B.** KDs were calculated from the curves fit to these data points and are included below.
