# Supplementary material for: A new class of antibodies that overcomes a steric barrier to cross-group neutralization of influenza viruses
Source: PLoS Biol. 2023 Dec 21;21(12):e3002415. doi: 10.1371/journal.pbio.3002415 (PMC10734940; doi:10.1371/journal.pbio.3002415)
Supplement: S4 Fig — (A) A top view of the HA RBS from the K03.28-HA complex. A sialic acid (abbreviated SIA) was modeled in from PDB 3UBE and is in yellow. Major antigenic determinants are colored on the structure and named in correspondingly colored text. Positions of 133a (red) and 226 (orange) are marked with spheres. (B) Sequence alignments of the antigenic surfaces surrounding the RBS (positions 127–229) for H3N2 and H1N1 HAs used in our binding studies. Identities to the reference sequence (top line of each alignment) are shown in dots, differences are shown in letters. Coloring of key features matches those used to color HA above in panel A. Sequence accession numbers: A/Aichi/02/1968(X-31)(H3N2) CY147438, A/Philippines/02/1982(H3N2)CY113301, A/Beijing/353/1989(H3N2) CY113301, A/Johannesburg/33/1994(H3N2) CY113301, A/Sydney/05/1997(H3N2) CY113301, A/Moscow/10/1999(H3N2), A/Fujian/411/2002(H3N2) CY112933, A/Wisconsin/67/2005(H3N2) ACF41911, A/Texas/50/2012(H3N2) KJ942616, A/Hong Kong/4801/2014(H3N2) KJ942616, A/Kansas/14/2017(H3N2) AVG71503, A/Tasmania/503/2020(IVR-221) EPI1752480, A/USSR/90/1977(H1N1) DQ508897, A/Chile/01/1983(H1N1) CY121261, A/Texas/36/1991(H1N1) CY033655, A/Solomon Islands/3/2006(H1N1) EU100724, A/California/04/2009(H1N1) FJ966082, A/California/07/2009(X-181)(H1N1) ACV82259, A/Michigan/45/2015(H1N1)EPI662594, A/Brisbane/02/2018(IVR-190)(H1N1)EPI1322979, A/Wisconsin/588/2019(H1N1) EPI1661758, A/Victoria/2570/2019(IVR-215)(H1N1) EPI1741926. (PDF) [file pbio.3002415.s005.pdf]

A

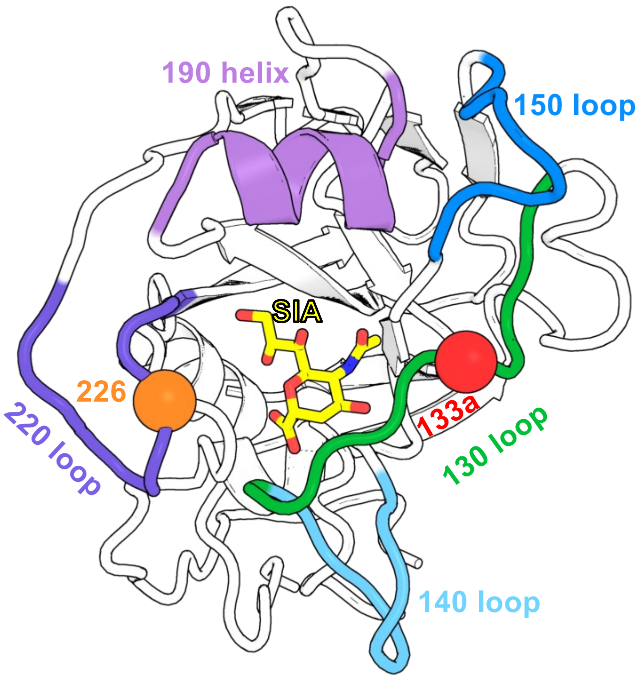

B

H3N2 HA receptor binding site

```
                                127                                                                                              226
A/Aichi/02/1968(X-31):          WTGVTQNGGSNACKRGPGSGFFSRLNWLTKSGSTYPVLNVTMPNNDNFDKLYIWGIHHPSTNQEQTSLYVQASGRVTVSTRRSQQTIIPNIGSRPWVRGLSSR
A/Philippines/02/1982:          ......S...ST....SNNS........YE.E.K...........GK..............DK...N..IR.........K.....V................
A/Beijing/353/1989:             ....A.S.E.Y.....SVKS........HE.EYK..A........GK........V.....DR...K...R.........K.....V................
A/Johannesburg/33/1994:         ....A.D.K.Y.....SVNS........H.LEYK..A........GK........V.....DSD......R.........K.....V..D..Y......Q...
A/Sydney/05/1997:               ....A...T.Y....SSIKS........HQLKYK..A.........K........V.....DSD...I.A..........K.....V............I...
A/Moscow/10/1999:               ....A...T.S....RSIKS........HQLENR..A.........K........V.....DSV...V............K.....V............V...
A/Fujian/411/2002:              ........T.S....RSNKS.........HLKYK..A........EK........VL..G.DSD.I...A.....I....K.....V........R...V...
A/Wisconsin/67/2005:            ........T.SS...RSNNS.........QLKFK..A........EK........V...V.DND.IF..A.....I....K.....V........RI.NIP..
A/Texas/50/2012:                .N......T.S..I.RSNNS.........HLNFK..A........EQ........V...V.DKD.IF..A.P...I....K....AV.....F..RI.NIP..
A/Hong Kong/4801/2014:          ........T.S..I.RSS.S.........HLNY...A........EQ........V...G.DKD.IF..A.S...I....K....AV........RI.DIP..
A/Kansas/14/2017:               .A......T.SS.I..SK.S.........HLN.K..A........EQ........V...G.DKD.I...A.S...I....K....AV........RI.DIP..
A/Tasmania/503/2020(IVR-221):   ....K...T.S..I..SS.S.........HLNYK..A.......KEQ........VY....DKD.I..FA.P...I....K....AV........RI..IP..
```

H1N1 HA receptor binding site

```
                                127    133a                                                                                       226
A/USSR/90/1977:                 WPKHNVTRGVTASCSHKGKSSFYRNLLWLTEKNGSYPNLSKSYVNNKEKEVLVLWGVHHPSNIEDQKTIYRKENAYVSVVSSNYNRRFTPEIAERPKVRGQAGR
A/Chile/01/1983:                .......K....A.....................................................................H..........K.....N.E..
A/Texas/36/1991:                ..N.T..K...T....N.................L............................R..RA..HT..........H.S........K.......E..
A/Solomon Islands/3/2006:       ..N.TT.-..S.....N.E....K......G...L........A................P..G..RAL.HT..........H.S.K......K.....DRE..
A/California/04/2009:           ..N.DSNK....A.P.A.AK...K..I..VK.GN...K.....I.D.G........I....TSA..QSL.QNADT..F.G..R.SKK.K....I.....D.E..
A/California/07/2009(X-181):    ..N.DSDK....A.P.A.AK...K..I..VK.GN...K.....I.D.G........I....TSA..QSL.QNAD...F.G..R.SKT.K....I.....DRE..
A/Michigan/45/2015:             ..N.DSNK....A.P.A.AK...K..I..VK.GN...K.NQ..I.D.G........I....TTA..QSL.QNAD...F.GT.R.SKK.K....T.....D.E..
A/Brisbane/02/2018(IVR-190):    ..N.DSNK....A.P.A.AK...K..I..VK.GN...K.NQT.I.D.G........I...PTTA..QSL.QNAD...F.GT.R.SKK.K....T.....DRE..
A/Wisconsin/588/2019:           ..N.DSDN....A.P.A.AK...K..I..VK.GK...KINQT.I.D.G........I...PT.A..QSL.QNAD...F.GT.R.SKK.K....T.....D.E..
A/Victoria/2570/2019(IVR-215):  ..N.DSDN....A.P.A.AK...K..I..VK.GK...KINQT.I.D.G........I...PT.A..QSL.QN.D...F.GT.R.SKK.K....T.....DRE..
```

S4. Antigenic variation surrounding the receptor binding site: A. A top view of the HA RBS from the K03.28-HA complex. A sialic acid (abbreviated SIA) was modeled in from PDB 3UBE and is in yellow. Major antigenic determinants are colored on the structure and named in correspondingly colored text. Positions of 133a (red) and 226 (orange) are marked with spheres. **B.** Sequence alignments of the antigenic surfaces surrounding the RBS (positions 127-229) for H3N2 and H1N1 HAs used in our binding studies. Identities to the reference sequence (top line of each alignment) are shown in dots, differences are shown in letters. Coloring of key features matches those used to color HA above in panel A. Sequence accession numbers: A/Aichi/02/1968(X-31)(H3N2) CY147438, A/Philippines/02/1982(H3N2)CY113301, A/Beijing/353/1989(H3N2) CY113301, A/Johannesburg/33/1994(H3N2) CY113301, A/Sydney/05/1997(H3N2) CY113301, A/Moscow/10/1999(H3N2),A/Fujian/411/2002(H3N2) CY112933, A/Wisconsin/67/2005(H3N2) ACF41911,A/Texas/50/2012(H3N2) KJ942616, A/Hong Kong/4801/2014(H3N2) KJ942616, A/Kansas/14/2017(H3N2) AVG71503, A/Tasmania/503/2020(IVR-221) EPI1752480, A/USSR/90/1977(H1N1) DQ508897, A/Chile/01/1983(H1N1) CY121261, A/Texas/36/1991(H1N1) CY033655, A/Solomon Islands/3/2006(H1N1) EU100724, A/California/04/2009(H1N1) FJ966082, A/California/07/2009(X-181)(H1N1) ACV82259, A/Michigan/45/2015(H1N1)EPI662594, A/Brisbane/02/2018(IVR-190)(H1N1)EPI1322979, A/Wisconsin/588/2019(H1N1) EPI1661758, A/Victoria/2570/2019(IVR-215)(H1N1) EPI1741926
